# Circulating Tumor Cells in Metastatic Breast Cancer: From Genome Instability to Metastasis

**DOI:** 10.3389/fmolb.2020.00134

**Published:** 2020-07-16

**Authors:** Ekaterina Ivanova, Ambber Ward, Adrian P. Wiegmans, Derek John Richard

**Affiliations:** ^1^Cancer and Ageing Research Program, Institute of Health and Biomedical Innovation, Queensland University of Technology, Translational Research Institute, Woolongabba, QLD, Australia; ^2^Centre for Tumour and Immune Biology (ZTI), Philipps University Marburg, Marburg, Germany; ^3^Tumor Microenvironment Laboratory, QIMR Berghofer, Herston, QLD, Australia

**Keywords:** liquid biopsy, circulating tumor cells, metastasis, genome instability, cell-free DNA

## Abstract

The emergence of clinical resistance in repeatedly treated cancers extends from the primary tumor's capability to exploit genome instability to adapt, escape, and progress. Triple negative breast cancer serves as a good example of such a response demonstrating poor clinical outcome due to a high rate of cellular heterogeneity resulting in metastatic relapse. The capability to effectively track the emergence of therapeutic resistance in real-time and adapt the clinical response is the holy grail for precision medicine and has yet to be realized. In this review we present liquid biopsy using CTCs and ctDNA as a potential replacement and/or addition to the current diagnostic tests to deliver personalized therapies to patients with advanced breast cancer. We outline current uses of liquid biopsy in the metastatic breast cancer setting and discuss their limitations. In addition, we provide a detailed overview of common genome instability events in patients with metastatic breast cancer and how these can be tracked using liquid biopsy.

## Introduction

Recurrence or metastasis following chemotherapy is a major clinical challenge in the treatment of cancer. Metastatic cancer cannot be cured using currently available treatment options and is responsible for 90% of cancer associated deaths (Guan, 2015). Consequently, there is a strong need to identify and eradicate cells capable of forming secondary tumors prior to them becoming re-established in the same or in a new location. Newly developed liquid biopsy technologies provide the potential to achieve this goal. Liquid biopsies are able to identify circulating tumor cells (CTCs) and cell free tumor products (e.g., circulating tumor DNA, cell free DNA, exosomes) that have escaped from the primary tumor, enabling molecular characterization, and the potential for clinicians to tailor precision medicine to the emergent therapy resistant cells.

Triple negative breast cancer (TNBC) serves as a good example of therapeutic challenge demonstrating poor survival due to a high rate of metastatic relapse (O'reilly et al., 2015; Park et al., 2018). Despite TNBC patients achieving higher pathologic complete response rates with chemotherapy compared to patients with other breast cancer subtypes, they have worse overall survival following chemotherapy than non-TNBC patients (Liedtke et al., 2008; Von Minckwitz et al., 2012; Cortazar et al., 2014; Haque et al., 2018; LeVasseur et al., 2020). If residual disease remains after neoadjuvant chemotherapy, TNBC patients are six times more likely to experience recurrence and 12 times more likely to die from metastatic disease (Brewster et al., 2014). Such adverse prognosis can in part be attributed to the lack of actionable cell surface targets like human epidermal growth factor 2 (HER2), estrogen receptor (ER), and progesterone receptor (PR) as well as molecular characteristics of the primary tumor that promote the development of chemotherapy resistant clonal variants, namely, genome instability (GI), and replication stress that drive a high degree of cellular heterogeneity (Chavez et al., 2010; Harbeck and Gnant, 2017; Park et al., 2018). Gene expression profile analysis of 21 breast cancer data sets revealed TNBC cellular heterogeneity clustered the into six molecular subtypes; basal-like (BL1 & BL20), immunomodulatory, mesenchymal, mesenchymal stem-like and luminal androgen receptor subtype (Lehmann et al., 2011). Although TNBC is a collection of essentially six different cancers, chemotherapy is still considered the standard of care for all patients. Under pressure of chemotherapy subclonal diversity within subtypes contributes to variability in responses and development of chemoresistance and metastasis (Zhang and Rosen, 2015). Patients may respond well initially to chemotherapy because the majority of cells in the tumor are sensitive to the drug. However, under the selective pressure of chemotherapy the rare chemoresistant cells survive and proliferate after treatment to cause recurrence (Kim et al., 2018). Using single-cell DNA and RNA sequencing in tumor samples collected from 20 TNBC patients during neoadjuvant chemotherapy, Kim et al. showed that clones with pre-existing genomic mutations and copy-number aberrations were initially adaptively selected by chemotherapy. Following adaptive selection, the surviving cells underwent transcriptional reprogramming as a result of chemotherapy to evolve the resistant phenotypes.

While recent therapeutic advances in treatment of hormone-positive or HER2-amplified metastatic breast cancers (MBC) demonstrate a significantly prolonged survival turning advanced metastatic cancer into a chronic disease, therapeutic pressure still drives intratumoural heterogeneity generating resistant phenotypes (Harbeck and Gnant, 2017). Changes in the biomarker status of metastases compared to the primary tumor are common in MBC, therefore, it is essential to identify molecular characteristics of metastatic lesions prior to commencing targeted therapy (Woo et al., 2019).

A number of proposed mechanisms involving failure to repair DNA damage, endogenous and oncogene-induced replication stress, telomere dysfunction have been described to fuel GI in cancer (Negrini et al., 2010). Breast cancer tumors display high levels of GI and an increased frequency of genetic alterations ranging from mutations in specific genes to general amplifications, insertions, deletions, or rearrangements even when unaccompanied with pressure from chemotherapy (Kalimutho et al., 2019). Whether these genotypes can be ascertained in CTCs or cell-free DNA (cfDNA) to accurately represent the diversity of the primary tumor is yet to be fully elucidated. Recent studies revealed that metastatic triple-negative breast cancers showed an increase in mutational burden including somatic biallelic loss-of-function mutations and enhanced clonal diversity compared to early triple-negative breast cancers (Bertucci et al., 2019). However, despite exhibiting increasing diversity, metastases are clonally related to the original primary cancer, sharing many of the driver mutations with emergence of acquired additional variants specific to metastasis (Yates et al., 2015). In this review we will discuss the role of liquid biopsy in diagnosis of metastatic breast cancer (MBC) progression, and the prognostic capability of CTCs and cfDNA based on analysis of specific phenotypic and genotypic markers.

## Liquid Biopsy

**Figure 1:**
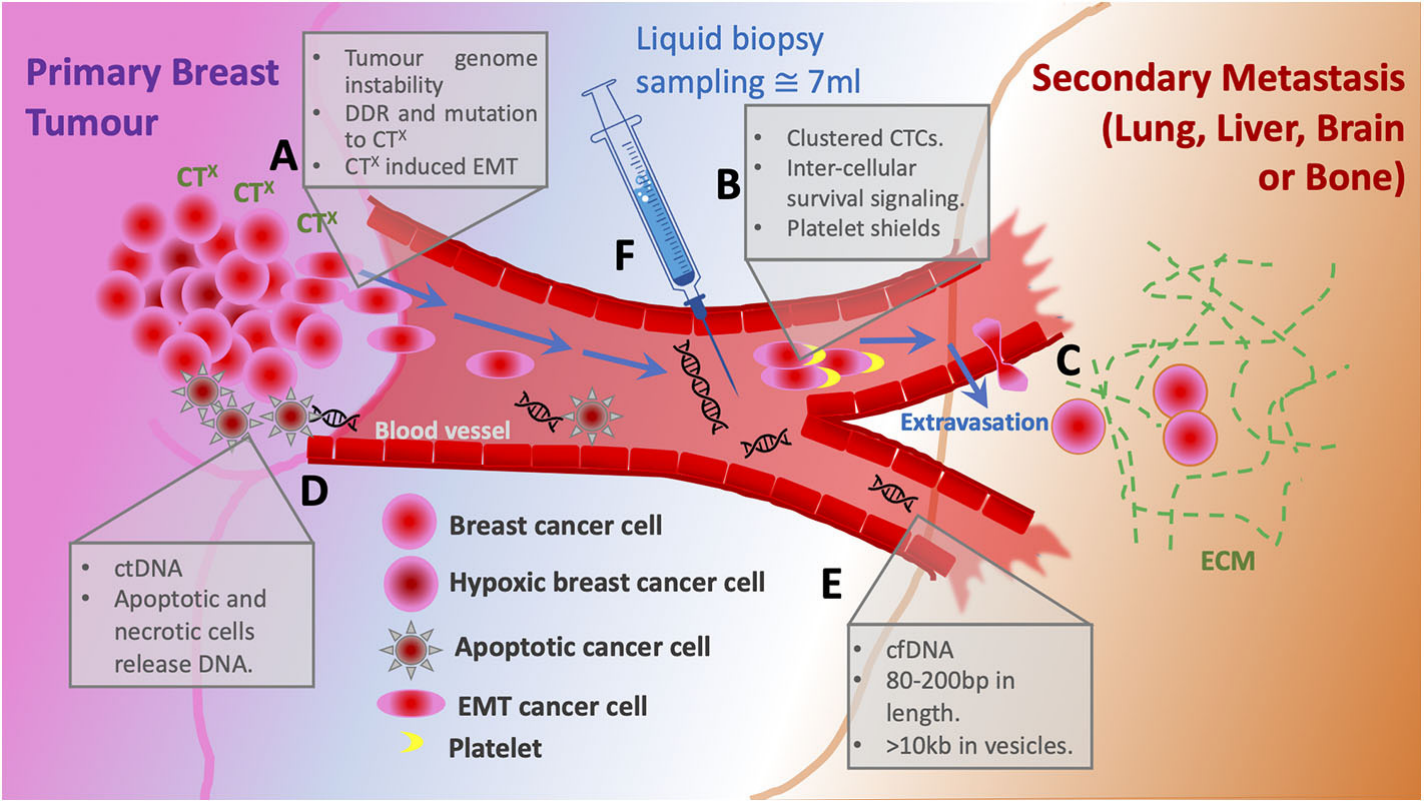
Schematic of metastatic progression of breast cancer and potential for liquid biopsy. **(A)** The primary tumor cancer cells harbor high levels of genome instability that under pressure from chemotherapy (CT^X^) respond with both enhanced DNA damage repair (DDR) and mutation rates. Altered cells acquire oncogenic phenotypes including EMT allowing single cell dissemination from the primary tumor. **(B)** Entering the bloodstream clustered circulating tumor cells bind platelets to evade immune response and gain survival signaling. **(C)** Circulating tumor cells extravasate to the secondary site supported by ECM. **(D)** Cells that die in response to CTx are the source of circulating DNA. **(E)** Cell free DNA is usually 80–200 bp, however when associated within a vesicle can be up to 10 kb. **(F)** Liquid biopsy of ~7 ml is taken from a patient to sample CTC numbers and cfDNA for sequencing.

Unlike conventional tissue biopsy, liquid biopsy is non-invasive, does not require the skills of highly trained medical personnel, can be performed as frequently as required and has only few adverse effects on patients. The growing interest of researchers in the technique is explained by its great potential to provide all-round patient-specific information—the clinical need which still has not been fully addressed. Liquid biopsy refers to obtaining and analyzing CTCs, circulating tumor nucleic acids [predominantly circulating tumor DNA (ctDNA)], and exosomes released into circulation by tumor cells (Figure 1). Already at early stages of cancer development tumor cells are shed into bloodstream by the primary tumor (Pantel and Alix-Panabières, 2019). More importantly, CTCs are precursors of metastatic lesions, as virtually all cells that eventually form metastases at distant sites will have undergone this transition (Figure 1A,C). CTC and ctDNA abundance in circulation is known to fluctuate in response to treatment (Diaz and Bardelli, 2014; Helissey et al., 2015), and with the use of liquid biopsies it is possible to assess treatment efficiency while in therapy or soon after therapy completion. The ease of obtaining material for analysis allows to draw serial blood samples within a short time frame, which in turn increases the chance of early detection of disease relapse. As discussed later in this review, ctDNA analysis not only provides accurate information about the presence of minimal residual disease, but also enables to detect clonal evolution of tumor cells and resulting new potentially actionable driver mutations. Similarly, detection and characterization of CTCs can give insights into tumor heterogeneity. In the era of personalized medicine liquid biopsy could facilitate a quicker transition to tailored targeted therapies by making diagnostic tests highly informative and more accessible. In this review we will focus primarily on CTCs and ctDNA and discuss their diagnostic and prognostic utility in MBC.

### CTCs

**Figure 2:**
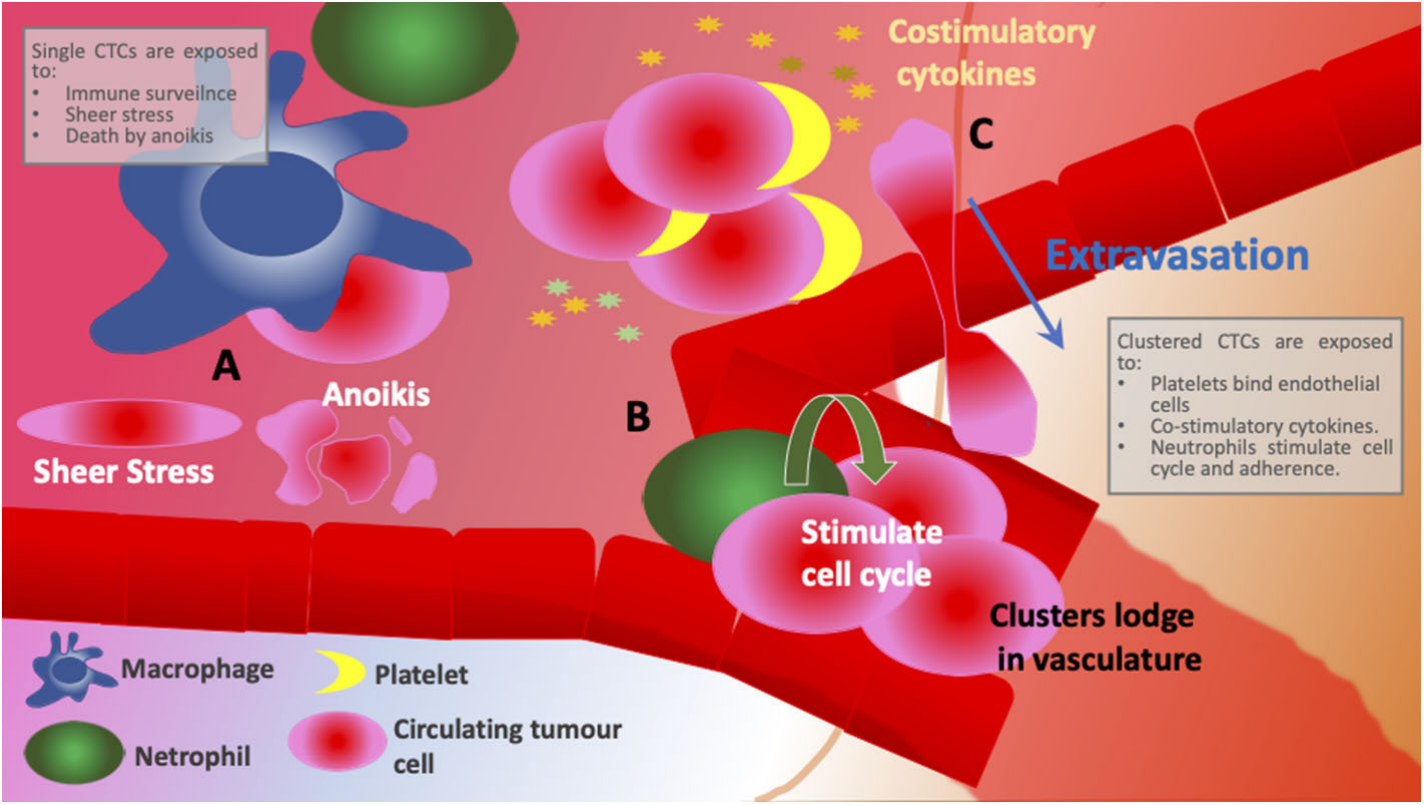
Schematic comparing different environments encountered between individual CTCs and clusters. **(A)** Individual CTCs are exposed to immune surveillance, shear stress and have low levels of adhesion and, therefore, open to cell death by anoikis. **(B)** CTC clusters are supported by neutrophil integration that induces cell cycle progression and DNA replication in CTCs. Larger cellular clusters are more likely to get entrapped in narrow vasculature promoting remodeling and secondary site growth. **(C)** Platelets interact with endothelial cells and anchor CTCs to the site of extravasation resulting not only in more efficient colonization at new sites but also in less time spent in circulation. CTC clusters provide co-stimulatory cytokines as well as corresponding cytokine receptors, evidence of immune reprogramming and active signaling.

CTCs are a truly unique subset of tumor cells. Firstly, the activation of the epithelial-to-mesenchymal-transition (EMT) program facilitates the intracellular “identity switch” which allows the cell to exit the site of primary tumor and enter the blood stream—a process termed intravasation (Figure 1A) (Kowalik et al., 2017). Secondly, upon entering the circulation, CTCs are exposed to a wide range of stresses including detection by immune cells, shear stress, loss of anchorage, and while most CTCs eventually succumb to any of these obstacles, some manage to survive in circulation (Figure 2A) (Mego et al., 2010; Kowalik et al., 2017). Lastly, CTCs arrive at a new site, where they extravasate and, given the right conditions, undergo mesenchymal-to-epithelial-transition (MET), and either become dormant to form a metastatic growth later or activate in a foreign microenvironment (Figure 1C) (Chambers et al., 2002). The entire process is inefficient as it requires cells to be very adaptable and possess a high degree of plasticity in order to constantly adjust to drastic changes in the environment (Massagué and Obenauf, 2016). Such resilience is a by-product of GI and the ever-increasing mutational burden accumulated naturally over time. Therapeutic exposure to DNA-damage-inducing agents (e.g., anthracyclines, platinum compounds, taxanes) exerts a tremendous pressure on the DNA damage response machinery which results in various accidental GI events (point mutations, insertions, deletions, chromosomal losses, and gains) and creates intratumoral heterogeneity (O'reilly et al., 2015; Harbeck and Gnant, 2017). The repeated use of cytotoxic drugs for cancers with an increased relapse potential is thought to enhance selection for resistant phenotypes with a high mutational burden, hence it is difficult to treat metastatic disease (Figure 1A) (O'reilly et al., 2015; Nedeljković and Damjanović, 2019).

As CTCs play such a crucial role in the establishment of metastasis, many researchers advocate for utilizing them in the clinical setting. CTC enumeration has served as a prognostic marker in MBC since the beginning of the twenty-first century, with CellSearch® being the first test system to be approved by the FDA for use in MBC. CellSearch® uses a cut-off of 5 CTCs per 7.5 ml of blood as a measure of poorer outcome (CELLSEARCH® | About CELLSEARCH® | Interpretation of Results^1^). This value was successfully validated back in 2004 (Cristofanilli et al., 2004), and a recent extensive review of data from 2,436 MBC patients from 17 European centers and the MD Anderson Cancer Center in the U.S. also deemed this cut-off appropriate (Cristofanilli et al., 2019). Moreover, it reported that patients, who were below the cut-off and, therefore, stratified as stage IV_indolent_ had a significantly longer overall survival of 37.1 months compared to stage IV_aggressive_ (≥5 CTCs per 7.5 ml. blood) with 15.4 months (Cristofanilli et al., 2019). Despite being FDA-approved and in clinical use for over 15 years, CellSearch® faces criticism in relation to its method of CTC detection. The test enriches EpCAM-positive, CD45-negative and cytokeratin 8, 18, and/or 19-positive cells i.e., cells of epithelial origin. Meanwhile, mounting evidence suggests that a subpopulation of CTCs undergoing EMT and, therefore, bearing less or no epithelial markers evade the detection by CellSearch® or any other EpCAM-based CTC enrichment method (Gorges et al., 2012; Moussavi-Harami et al., 2014; Mitra et al., 2015; de Wit et al., 2018; Papadaki et al., 2019). Indeed, in the breast cancer setting a significant downregulation of EpCAM expression goes hand in hand with cell detachment and subsequent migration both of which are signs of EMT (Rao et al., 2005; Kyung-A Hyun et al., 2016). Activated EMT and cancer stem cell (CSC) programs have long been associated with greater risk of resistance to therapy, disease relapse, and poorer survival (Prieto-Vila et al., 2017; Williams et al., 2019). More importantly, some evidence indicates that active EMT contributes to survival of CTCs in circulation and, as a result, these cells are more successful at forming metastases (Bonnomet et al., 2010; Agnoletto et al., 2019). Interestingly, Kaigorodova and colleagues reported that EpCAM-negative CTC numbers increased after repeated exposure to neoadjuvant chemotherapy (Kaigorodova et al., 2018). Based on the aforementioned facts, it could be argued that accurate detection of all CTCs is crucial, and a better CTC capture platform is necessary (Table 1), as CTCs that go undetected by current technologies approved for clinical use are the ones posing the greatest risk of cancer spread. Nevertheless, it is still unclear, whether the presence of EMT-active CTCs in blood is a risk stratification marker (Tsai and Yang, 2013; de Wit et al., 2018), whereas enumeration of EpCAM-expressing CTCs has been validated and proven to be prognostically highly relevant. All in all, it is evident that more research into the biology of CTCs is necessary in order to provide a better understanding of the clinical significance of EMT and CSC phenotypes in CTCs.

**Table 1:** A summary of methods for CTC detection and ctDNA analysis.

**Source material**	**Method**	**Advantages and/or limitations**	**Clinical use**	**References**
CTC	ApoStream®	CTC isolation based on cells' biophysical properties (volume, surface area, density, protein content, conductivity)	CTC detection, molecular characterization	Gupta et al., 2012
	CellSearch®	FDA approved, validated prognostic performance; detects EpCAM-expressing CTCs only	CTC enumeration for diagnosis, isolation for molecular characterization	Moussavi-Harami et al., 2014; Cristofanilli et al., 2019
	Cytophone	Detection of CTCs and CTC clusters in blood using *in vivo* photoacoustic flow cytometry, does not require blood collection, developed for use in melanoma	CTC and CTC cluster detection, possible CTC neutralization directly in blood	Galanzha et al., 2019
	Cytospin™	CTC detection in a pool of peripheral blood mononucleated cells (PBMCs), no enrichment/isolation steps	Marker-dependent detection of CTCs, molecular characterization	Agelaki et al., 2015, 2017; Papadaki et al., 2019
	Diagnostic LeukApheresis	Potentially increases sensitivity of CTC detection	Leukocyte depletion prior to CTC detection	Andree et al., 2018
	ISET®	CTC isolation by size, no cell surface markers used	CTC detection, molecular characterization	Vona et al., 2000; Farace et al., 2011
ctDNA	Bisulfite Conversion	Allows to locate and analyze methylated regions of ctDNA by methylation specific PCR or sequencing	Detection of resistance-related methylation signatures.	Matuschek et al., 2010; Sharma et al., 2010a; Mastoraki et al., 2018
	Digital PCR (ddPCR)	Detection at very low concentrations	ctDNA detection, screening for known mutations and copy number variations associated with resistance	Murtaza et al., 2013; Schiavon et al., 2015; Siravegna et al., 2017; Sakai et al., 2018
	Target-Capture Sequencing/Targeted Amplicon Sequencing	Allows to identify and analyze novel mutations	Detection of mutations and copy number variations in genes known to be implicated in emergence of resistance	Cristofanilli et al., 2013; Murtaza et al., 2013; Guttery et al., 2015; Ma et al., 2016; Weigelt et al., 2017; Lin et al., 2019
	Whole Exome Sequencing	Identification of all changes in coding ctDNA sequence compared to somatic non-tumor DNA, comprehensive analysis	Detection and characterization of novel resistance-associated genes	Siravegna et al., 2017
	Whole Genome Sequencing	Analysis of chromosomal aberrations and rearrangements with ctDNA	Detection of cancer-related chromosomal aberrations and affected genes	Leary et al., 2012

*Rostami et al. offer a more comprehensive overview of current CTC detection platforms (Rostami et al., 2019)*.

#### CTC Biology

CTCs are potentially a powerful tool to track phenotypical changes, tumor evolution and response to treatment. In a study by Agelaki et al. it was shown that not only metastatic TNBC patients but also those at early disease stage may have ER-, PR-, and HER2-expressing CTCs (Agelaki et al., 2017). Moreover, in early TNBC patients there was a significant decrease in hormone receptor and HER2-positive CTCs accompanied by a distinct increase in EGFR-expressing CTCs after adjuvant chemotherapy. In TNBC patients with metastatic disease HER2-positive CTCs but not hormone receptor expressing CTCs were detected at higher frequency compared to early stage patients. Reactivation of HER2 represents a positive marker for metastatic progression in particularly breast-to-brain metastases (Witzel et al., 2018). Interestingly in a model of breast to brain metastasis GI was found to be essential and induced via reactive oxygen species in the metastatic neuro-inflammatory microenvironment (Woditschka et al., 2014). Therefore, plasticity of CTCs driven by GI can be considered an essential phenotype for successful metastatic progression.

Association of CTCs with activated platelets in bloodstream is thought not only to provide pro-survival signals (Figure 2C) and shield from detection by immune cells, it also facilitates extravasation whereby platelets interact with endothelial cells and anchor CTCs to the site of extravasation (Figure 1B) (O'Flaherty et al., 2012; Yu et al., 2013; Aceto et al., 2014; Heeke et al., 2019). In contrast to the hypothesis that CTCs downregulate metabolism and most essential survival pathways, a recent study by Szczerba et al. reported that neutrophils supported cell cycle progression and DNA replication in CTCs by forming neutrophil-CTC clusters (Figure 2B) (Szczerba et al., 2019). Furthermore, cells within CTC-neutrophil clusters expressed co-stimulatory cytokines as well as corresponding cytokine receptors—an evidence of immune reprogramming and active signaling (Figure 2B). The group also found that patients with at least one neutrophil-CTC cluster per 7.5 ml blood had a significantly shorter progression-free survival than patients with five or more CTCs per 7.5 ml blood (a cut-off used by CellSearch® for unfavorable prognosis). Immune evasion, facilitated by the crosstalk between CTCs, CTC clusters and immune cells, is a prerequisite for successful metastatic dissemination (Heeke et al., 2019). Hence, inhibition of CTC-immune cell/platelet cluster formation could hold promise as therapeutic strategy (Choi et al., 2015; Heeke et al., 2019).

Clusters of CTCs or tumor microemboli are posited to have a higher metastatic potential than single CTCs (Aceto et al., 2014; Hong et al., 2016; Giuliano et al., 2018; Rostami et al., 2019). Compared to single CTCs, aggregations of carcinoma cells have survival advantages. Firstly, CTCs within clusters express higher levels of cell adhesion molecules compared to single CTCs which allows them to retain anchorage to neighboring cells and, therefore, escape cell death by anoikis (Aceto et al., 2014; Cheung et al., 2016; Giuliano et al., 2018; Wei et al., 2018). Secondly, by forming a cluster, cells protect each other from a number of stresses which single CTCs are exposed to including shear stress and immune surveillance (Cheung et al., 2016; Rostami et al., 2019). Finally, larger cellular clusters are more likely to get entrapped in narrow vasculature resulting not only in more efficient colonization at new sites but also in less time spent in circulation (Peeters et al., 2015; Hong et al., 2016). Indeed, in lungs which are a common site of metastasis in breast cancer, mesh-like vasculature seems to act as a sieve and retains CTC clusters in patients with MBC (Peeters et al., 2015). A similar observation was made in mouse models of breast cancer cell dissemination (Aceto et al., 2014; Cheung et al., 2016). As a result, it comes as no surprise that the presence of CTC clusters in circulation correlates with poor prognosis in MBC patients (Aceto et al., 2014; Mu et al., 2015; Wang et al., 2017a). Furthermore, clustered CTCs possess a characteristic DNA-hypomethylation pattern which is associated with increased proliferation and enhanced stemness phenotype (Gkountela et al., 2019). This methylation signature also seems to be indicative of poorer outcome in breast cancer patients (Gkountela et al., 2019). Interestingly, upon cluster dissociation some of the identified hypomethylated regions of DNA gained methylation (Gkountela et al., 2019), suggesting that inhibition of CTC cluster formation could have therapeutic value. Although we are yet to fully elucidate the biology of CTCs, there is growing evidence for their utility in the clinic as both diagnostic markers of potential metastasis and prognostic markers of outcome.

### Circulating Tumor DNA

A liquid biopsy also has the option to isolate and analyze nucleic acids. Circulating nucleic acids comprises the fraction of circulating cell-free DNA/RNA originating either the primary or metastatic tumors. This includes short nucleosome-associated fragments (80–200 bp) or longer fragments (>10 kb) encapsulated within extracellular vesicles (Figure 1E) (De Rubis et al., 2019). The mechanisms of ctDNA release into circulation include cell death; apoptosis, necrosis, lysis of CTCs, and active secretion from the tumor (Stroun et al., 2006). The obvious advantage of ctDNA over CTCs is the relative ease of ctDNA capture and enrichment. There are several highly accurate and sensitive molecular detection methods; such as droplet digital PCR (ddPCR) and next generation sequencing (NGS), which are currently the go-to tools for ctDNA analysis (Pantel and Alix-Panabières, 2019; Zhang et al., 2019). NGS offers a comprehensive overview of all genetic alterations, allowing for the discovery of new unique mutations which result from the evolution of tumor cells under therapy-induced selective pressure. The timely identification of such changes in specific genetic loci plays a crucial role in diagnosis and treatment decisions (Schiavon et al., 2015; Weigelt et al., 2017). For instance, breast cancer patients carrying germline *BRCA1/2* mutations initially present with tumors lacking functional HR DNA repair pathway. Later, these patients often develop post-therapy metastases with restored HR function due to a phenomenon termed “*BRCA* reversal,” thus, the few cancer cells which were able to withstand DNA damaging agents and/or PARP inhibition form metastatic growths (Bouwman and Jonkers, 2014; Johnson et al., 2014). Several studies have shown that it is, indeed, possible to identify *BRCA* reversion (resistant cells) shortly after completing a round of therapy (Weigelt et al., 2017; Lin et al., 2019). Moreover, the analysis of ctDNA also revealed that multiple unique mutations within *BRCA1/2* genes lead to the restoration of the reading frame—an evidence that not only suggests a polyclonal nature of this particular resistance mechanism, but also underlines the significance of multiclonal heterogeneity in advanced cancers. Weigelt and colleagues identified *BRCA2* reversal mutations in cfDNA of a patient who had just completed treatment with carboplatin. Later, this very patient did not respond to therapy with a PARP inhibitor talazoparib, confirming the prognostic capability of cfDNA (Weigelt et al., 2017).

## Circulating Oncogene DNA: Diagnostic and Prognostic Utility

In estrogen receptor (ER) positive breast cancer patients, prolonged exposure of tumor cells to endocrine therapy is known to eventuate in resistant metastatic lesions harboring mutations in the *ESR1* gene and poor patient outcome (Schiavon et al., 2015; Lei et al., 2019). Therefore, identifying the mechanisms underlying progression to a resistant phenotype is vital. To this day, several groups have reported the clinical feasibility of detecting *ESR1* mutations in cfDNA (Guttery et al., 2015; Schiavon et al., 2015; Chu et al., 2016). These studies not only validated the use of cfDNA for diagnostic and prognostic purposes but also highlight the advantage of ctDNA/cfDNA liquid biopsy over tissue biopsy of sites of metastases. Specifically, in the study led by Chu et al. analysis of ctDNA in some patients identified additional *ESR1* mutations distinct from those identified in metastatic lesions, highlighting possible future or established micrometastases not been present and/or known of at the time of tissue biopsy.

Another example of diagnostic and prognostic potential of ctDNA is in the acquired resistance to anti-HER2 therapy which is characterized mainly by the following molecular mechanisms: downstream activation of PI3K signaling pathway (e.g., activating mutations in PI3K catalytic subunit, loss of functional tumor suppressor PTEN) and expression of constitutively active truncated p95-HER2 receptor lacking trastuzumab binding site (Gajria and Chandarlapaty, 2011). This results in an increased compensatory reliance on other facilitators of growth signaling such as ER, progesterone receptor (PR), and insulin receptor (IR), and, finally, loss of *ERBB2* (Gajria and Chandarlapaty, 2011; Sakai et al., 2018; Branco et al., 2019). The latter is especially common in hormone receptor positive tumors (Sakai et al., 2018; Branco et al., 2019). Loss of *ERBB2* amplification was successfully diagnosed in patient ctDNA obtained prior to treatment with trastuzumab emtansine (T-DM1)—an antibody-drug (maytansinoid) conjugate, and was associated with primary resistance to HER2-targeted therapy (Sakai et al., 2018). Likewise, monitoring the temporal dynamics in the presence of *ERBB2* amplification in circulating DNA was reported to be predictive not only of resistance to anti-HER2 therapy, but also of disease dormancy and progression (Ma et al., 2016). In one exemplary case, Ma et al. observed a steady increase in *ERBB2* gene copy number after the fourth cycle of therapy and 8 weeks prior to the clinical manifestation of disease recurrence (Ma et al., 2016). In addition, compelling results show the feasibility of liquid biopsy for *ERBB2* amplification with ctDNA from cerebrospinal fluid to identify metastatic disease to the brain (Siravegna et al., 2017). Markedly, HER2 activating mutations may arise in metastasized cells as a resistance mechanism in *ERBB2* non-amplified breast cancer (Wang et al., 2017b). Depending on the nature of such altered HER2 signaling, patients could benefit from targeting HER2 to treat metastases and, as such, ctDNA analysis could track evolution of the molecular characteristics of multiple metastatic lesions (Ma et al., 2017). This type of diagnosis is the basis of precision medicine and can be applied to other genes. Mutations in *PIK3CA* and other members of PI3K downstream signaling such as PTEN and mTOR, are some of the most frequently reported aberrations in breast cancer (Zardavas et al., 2014). Hence, it is not suprising that mutated *PIK3CA* and *MTOR* were identified in ctDNA and associated with resistance (Murtaza et al., 2013; Ma et al., 2016, 2017; Kodahl et al., 2018; Sakai et al., 2018). Importantly the ratio of mutated *PIK3CA* along with *TP53* can be used as a laboratory biomarker to differentiate ctDNA from cfDNA that is not tumor derived, therefore reducing the potential for false positives (Cristofanilli et al., 2013; Diaz and Bardelli, 2014; Schiavon et al., 2015).

### Alternative Serum Markers

Initially, research into serum biomarkers revealed the utility of non-nucleic acid markers. Cancer antigen 15-3 (CA 15-3) was discovered a potential serum biomarker associated with breast cancer metastasis, however, some chronic diseases like liver cirrhosis, sarcoidosis, hypothyroidism and megablastic anemia are also known to elevate CA 15-3 levels (Duffy et al., 2010). Furthermore, the study conducted by Dawson et al. demonstrated a superior sensitivity of ctDNA quantification (96%) in identification of patients with metastases compared to CA 15-3 measurement (78%) (Cristofanilli et al., 2013). They also reported that ctDNA had been found to better reflect dynamic changes in tumor burden, treatment response and that ctDNA was an excellent measure of clonal heterogeneity in the tumor. In a proof-of-principle study, Leary et al. performed whole-genome sequencing on ctDNA of colorectal and breast cancer patients to identify and analyze chromosomal rearrangements (Leary et al., 2012). Despite a rather small sample size (only three breast cancer-related samples), the group reported intra- and interchromosomal changes to chromosomes 1, 7, 11, and 13 affecting *CAMK1G, CDK6*, and *STK24* genes. This was an early example of GI driving future metastases.

Aberrant methylation goes hand in hand with cancer development (Łuczak and Jagodzinski, 2006). As evidenced by studies whose results are discussed below, bisulphite conversion is a common technique in epigenetics that allows to identify methylated regions of DNA. One of the most common resistance mechanisms that tumor cells utilize is by simply pumping cytotoxic drugs out of the cell. This process is facilitated by ATP-binding cassette (ABC) transporters—a family of transmembrane proteins which utilize ATP hydrolysis to transport biomolecules across the cell membrane (Wilkens, 2015). It was found that promoter hypomethylation of multidrug resistance 1 (*MDR1*) gene, which encodes P-glycoprotein (P-gp)—a member of the ABC transporter family, is linked to an increased expression of P-gp, which, in turn, correlates with resistance and poor survival (Sharma et al., 2010a; Besse et al., 2018). Sharma et al. successfully detected *MDR1* promoter hypomethylation in patient plasma DNA and were able to match the *MDR1* hypomethylation status to that in tumor tissue samples. Moreover, the group conducted a similar study focusing on hypermethylation of some key DNA maintenance genes such as *MGMT* and *BRCA1* and, similarly, hypermethylation of these genes in plasma, was concordant with their hypermethylation status in tumor tissue (Sharma et al., 2010b). Increased methylation of important tumor suppressor genes adenomatous polyposis coli (*APC*) and *RASSF1* was found to be implicated in breast cancer metastases (Matuschek et al., 2010). As well as being prognostic for metastases, methylation was found to be diagnostic for therapy resistance. *ESR1* promoter hypermethylation was identified in CTC DNA and paired ctDNA samples of patients with ER-positive/HER2-negative advanced breast cancer, who failed to respond to a combined treatment with mTOR and aromatase inhibitors (Mastoraki et al., 2018). More importantly, results of ctDNA analysis were highly concordant with those of CTC-derived DNA methylation analysis (>95%), implying that ctDNA could, indeed, be utilized independently for diagnostic testing to produce reliable results. By employing powerful analytical tools such as high coverage sequencing with subsequent bioinformatical analysis, it is feasible not only to identify single epigenetic markers but also to generate stratified diagnostic and/or predictive methylation patterns (Widschwendter et al., 2017). However, high amounts of patient data are required to formulate the patterns, and, in addition to this, heterogeneity of MBC is likely to complicate this undertaking. It should also be noted that the high cost of such extensive ctDNA analysis makes it currently clinically impractical.

## Precision Medicine Based on Liquid Biopsy Prognosis

**Figure 3:**
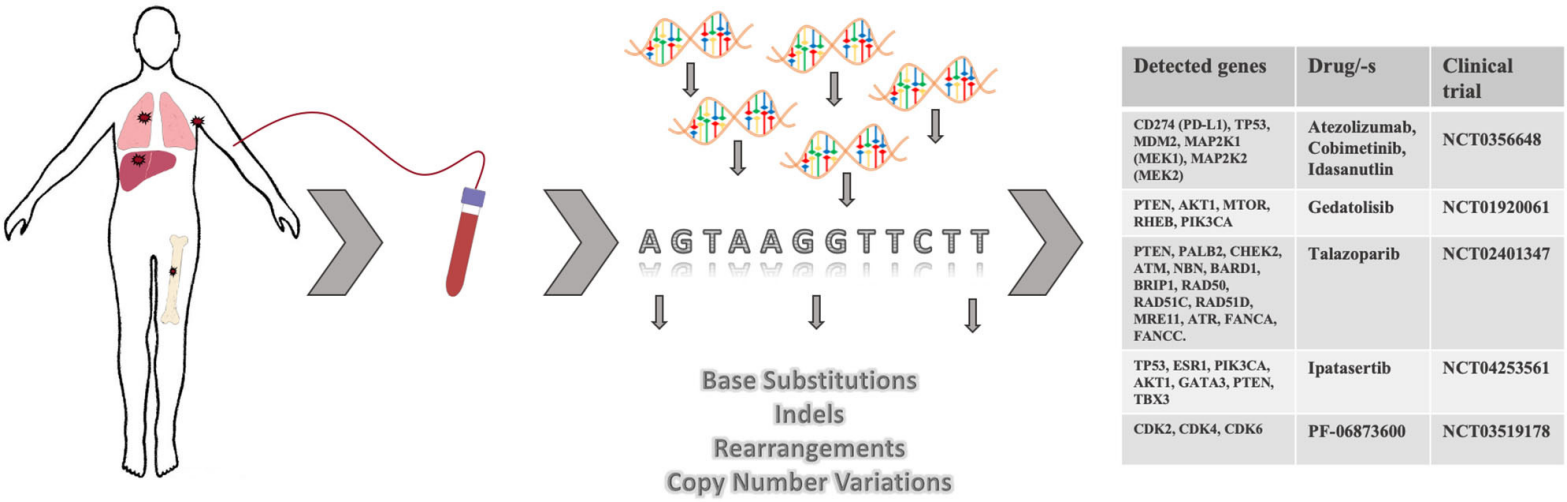
Targeted therapies based on ctDNA analysis and identification of actionable mutations in metastases. Upon discovery of minimal residual disease or at radiological evidence of metastatic spread, patient's blood may be collected for ctDNA analysis. Based on the identified mutations in ctDNA, patients may be referred to a clinical study where a drug/-s targeting actionable mutations is/are utilized.

In the past decade the medical community has contributed to and witnessed the development of more informative and reliable liquid biopsy-based clinical tests. Not that long ago the idea of monitoring cancer progression through a simple blood draw only seemed futuristic and ambitious. Nowadays, for some solid cancers including metastatic breast cancer, CTC enumeration or ctDNA analysis have become not just a routine test for prognostic purposes but include diagnostic potential (Figure 1F). By conducting genetic profiling of ctDNA, patients with advanced metastatic breast cancer have an opportunity to receive more personalized therapies exploiting genetic aberrations in distant metastases, rather than just another round of chemotherapy (Figure 3). As an example of this, Foundation Medicine has created a platform that incorporates the results of their ctDNA liquid biopsy FoundationOne® Liquid analysis to identify unique mutations and match patients with relevant clinical trials (FoundationSmartTrials™ | Foundation Medicine^2^). A similar service is offered by Guardant Health the developer of Guardant360® ctDNA liquid biopsy test. As of April 2020, both biotech companies are seeking FDA approval for their test platforms.

It is very likely that liquid biopsy may eventually replace serum biomarkers as diagnostic tests. CTC and ctDNA detection demonstrated superior sensitivity and accuracy in monitoring breast cancer progression than serum biomarker CA 15-3 (Cristofanilli et al., 2013; Bidard et al., 2014). Notably, measuring levels of serum biomarkers has a limited informative value compared to liquid biopsy as both CTCs and ctDNA can be characterized comprehensively. Moreover, protein biomarkers are known to remain in circulation for weeks and can be affected by many factors potentially producing misleading results. In contrast, ctDNA is estimated to have a short half-life of ~2 h (Diaz and Bardelli, 2014). This allows to monitor initial response to therapy and, if required, adjust the regimen early on, without the need to wait for radiological signs of disease progression.

When it comes to comparing CTCs and ctDNA, there seems to be little consensus which source material is better. As a matter of fact, most studies focus on either one biopsy marker exclusively and, therefore, do not present a well-balanced, unbiased assessment. Madic et al. claim in their study that unlike with CTC numbers they could not establish a prognostically relevant correlation between ctDNA levels and time to progression and overall survival in metastatic TNBC (Madic et al., 2015). On the contrary, Dawson and colleagues found ctDNA detection to be more sensitive, specific, and reflective of changes in tumor burden than CTCs (Cristofanilli et al., 2013). It could be argued that by utilizing highly sensitive methods like digital PCR and NGS (Table 1)—followed by an appropriate bioinformatical analysis, ctDNA could provide versatile and robust information even with very little starting material. Nevertheless, special consideration should be taken when analyzing ctDNA while undergoing therapy, as apoptotic tumor cells are believed to be the primary source of ctDNA (Figure 1D) and its increased abundance could interfere with genetic profiling of rare cells with emerging resistance (Diaz and Bardelli, 2014; Pantel and Alix-Panabières, 2019). However, for this very reason an increase in ctDNA during therapy could be interpreted as an indicator of response to therapy.

CellSearch® remains the most commonly used CTC enrichment method for research purposes, possibly owing to it being FDA-approved. As discussed in this review, CellSearch® fails to enrich EpCAM-low/negative CTCs, which highlights the need for a more sensitive and robust CTC detection method. A better CTC capture platform (Table 1) would, without a doubt, facilitate better understanding of CTC biology by allowing access to more CTC subtypes. As CTCs offer a wide range of analytes, improved capture together with advances in methods of single cell analysis would also increase our knowledge of CTC proteome and metabolome which could possibly help to elucidate mechanisms of metastasis even further.

As the debate over methods of CTC detection continues, we believe that ctDNA is currently the most suitable source material to track and analyze GI events during cancer progression. Emergence of re-activating *BRCA1/2* mutations, activating *ESR1* and *ERBB2* mutations, loss of *ERBB2* amplification as well as chromosomal rearrangements are prime examples of GI as a mechanism of therapy resistance in progressing tumors. As described in this review, these events can be detected in ctDNA. Although, some methods presented in this review are not available commercially as diagnostic tests yet (Table 1), it is possible that the growing public interest in liquid biopsy will attract more funding into the industry and facilitate the development of such tests.

## Author Contributions

EI conceived the idea for the review, conducted literature research, wrote the text, and made Table 1 and Figure 3. AW and APW reviewed the manuscript, provided critical feedback, and assisted with Figures 1, 2. DR reviewed the final draft. All authors contributed to the article and approved the submitted version.

## Conflict of Interest

The authors declare that the research was conducted in the absence of any commercial or financial relationships that could be construed as a potential conflict of interest.

## Footnotes

^1^CELLSEARCH® | About CELLSEARCH® | Interpretation of Results. Available online at: https://www.cellsearchctc.com/clinical-applications/interpretation-of-results (accessed March 1, 2020).

^2^FoundationSmartTrials™ | Foundation Medicine. Available online at: https://www.foundationmedicine.com/insights-and-trials/foundation-smarttrials#enrollment (accessed April 5, 2020).
